# Orbital Floor and Maxillary Reconstruction With Titanium Mesh and Anterolateral Thigh Free Flap

**Published:** 2019-07-11

**Authors:** Paul Lavadera, Jerette Schultz, Jeremy C. Sinkin

**Affiliations:** ^a^Division of Plastic and Reconstructive Surgery, Department of Surgery, Robert Wood Johnson Medical School, New Brunswick, NJ; ^b^Division of Plastic and Reconstructive Surgery, Department of Surgery, Rutgers New Jersey Medical School, Newark

**Keywords:** anterolateral thigh flap, maxillary reconstruction, maxillectomy, fibrous dysplasia, orbital floor

## Abstract

A 57-year-old woman with a 15-year history of a slowly growing fibrous dysplastic maxillary bony tumor underwent total left maxillectomy with subsequent maxillary reconstruction with anterolateral thigh single perforator free flap and orbital floor reconstruction with a titanium mesh implant.

## CASE DESCRIPTION

**Figure 1:**
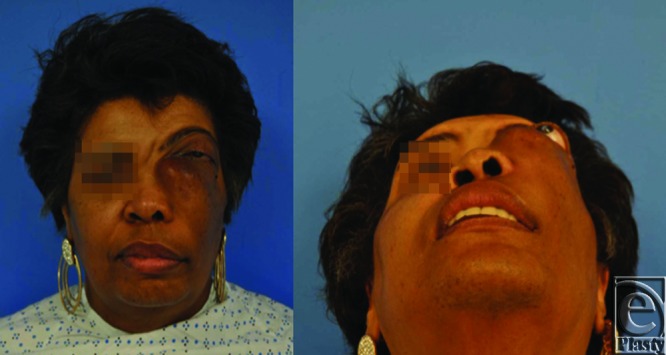
Preoperative photographs showing significant proptosis and distortion of the patient's left orbit.

**Figure 2:**
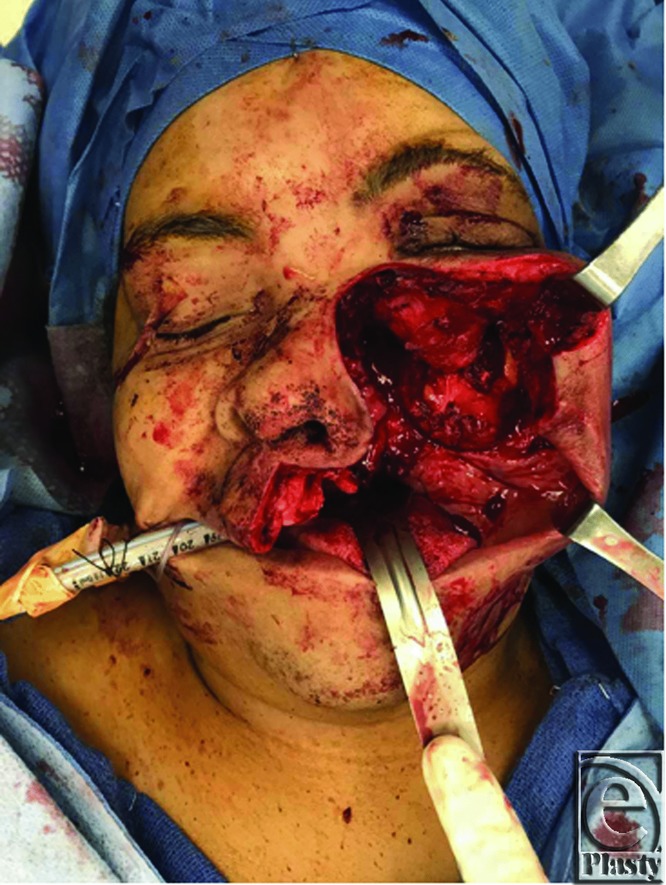
Intraoperative defect after tumor resection.

**Figure 3:**
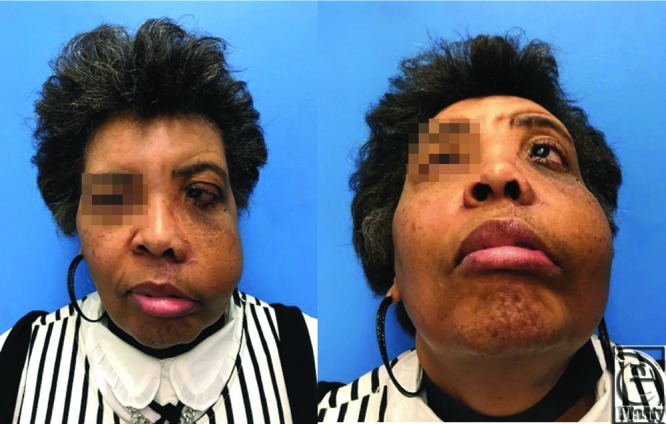
Postoperative photographs.

A 57-year-old woman presented with a 15-year history of a slowly growing left maxillary bony tumor causing significant proptosis and distortion of her left orbit with associated loss of vision (Fig 1). The patient underwent resection by ENT via a modified Weber-Ferguson incision (Fig 2).^1^ The resulting defect included the left hemi hard palate, lateral nasal wall, orbital floor, and lateral wall of the maxilla. The orbital floor was reconstructed with a titanium mesh implant and the soft tissue was reconstructed with an anterolateral thigh single perforator free flap. The skin paddle was folded to reconstruct the intraoral palatal defect and lateral intranasal wall. The final pathology report showed an 8 cm long fibro-osseous lesion with features suggestive of fibrous dysplasia. Postoperatively, the patient has improved globe position and has resumed a normal diet with her dentures. The patient will undergo revision surgery including flap debulking and soft tissue resuspension (Fig 3).

## QUESTIONS

1. What are the material options for reconstruction of the orbital floor after orbital-content sparing total maxillectomy?
2. What are the options for soft tissue reconstruction after total maxillectomy?
3. What is fibrous dysplasia?
4. What are the treatment options for fibrous dysplasia tumors?

## DISCUSSION

Numerous materials, both alloplastic and autogenous, are available for reconstructing damaged or resected orbital walls to restore orbital structure and volume. Autogenous calvarial bone graft has traditionally been the standard material used and shown to have a slight advantage in donor site morbidity over the use of rib and ilium.^2^ Autogenous graft is recommended when adjuvant radiation therapy is necessary. Alloplasts such as polyethylene and titanium meshes have gained popularity for orbital wall reconstruction for their ease of use and the elimination of the donor site and its associated morbidity. Titanium mesh has good biocompatibility, is visualized on computed tomographic scans with little artifact, and is rigid though easily adjustable to be trimmed and molded exactly to the orbital contour to prevent sagging and displacement into the maxillary antrum. The titanium mesh structure allows for connective tissue to grow around and through the implant to prevent its migration.^3^ However, subsequent orbital trauma may displace a mesh and endanger the optic nerve, which is not encountered with calvarial bone grafts.^3^ Titanium mesh plates may exhibit an advantage over resorbable mesh plates, which report to produce inflammatory reactions and fibrotic bands leading to gaze restriction in patients.^4^ Porous polyethylene mesh can be used in small (<2 cm), linear defects without gross comminution. However, polyethylene mesh is not radiopaque and cannot be easily visualized on immediate postoperative scans. A study from 2009 assessed the autogenous (calvarial bone graft) and alloplastic options (titanium and mesh) used in 10 patients with orbital floor reconstruction after traumatic maxillary fracture.^3^ The article concluded that all 3 materials were found to be satisfactory postoperatively and the ideal material for reconstruction is influenced by factors including characteristics of the injury, cost, patient choice, and experience and opinion of the surgeon.

The use of local, regional, or distant free flaps for soft tissue reconstruction after total maxillectomy can result in good function and aesthetics. Free flaps are commonly used; however, a temporalis muscle flap may be used for small- to medium-sized palate defects.^5^ The temporalis harvest may be combined with an approach often needed to expose the infratemporal fossa. After harvest through an ipsilateral hemicoronal incision, the flap can be passed into the maxillectomy or palatectomy cavity after removal of the zygomatic arch. Alternatively, the choice of autologous tissue selection varies on the vascular condition of the donor site, flap surface-to-volume ratio, pliability of tissues, and pedicle size and length. Radial forearm fasciocutaneous flaps satisfy many of these criteria and are commonly used for small volume defects. Other donor sites, including the rectus abdominus, lateral arm, and serratus anterior muscle, have also been successfully applied to reconstruct these defects. Large-volume maxillectomy defects typically require microvascular reconstruction using anterolateral thigh flaps. Commonly, multiple skin paddle reconstruction is required to recreate palatal or external skin defects. However, use of additional skin paddle for mucosal lining is often not necessary as vascularized fascia of the island perforator flap will granulate intraorally or intranasally and will mucosalize without the use of additional paddles. The large-volume musculocutaneous flaps may suffer from lack of support, which results in soft tissue ptosis into the oral cavity and osteocutenous flaps can be used for better support. Techniques including suture suspension techniques and manipulation of the fascia can be done to prevent intraoral flap prolapse of soft tissue flaps.

Fibrous dysplasia is a noninherited developmental anomaly of bone where bone marrow in the medullary cavity is replaced by fibrous connective tissue and poorly formed trabecular bone.^6^ This is caused by the somatic mutation in the guanine nucleotide stimulatory protein (*GNAS1*) gene, which encodes the alpha subunit of the stimulatory G protein.^7^ The tumor can be localized to a single bone (monostotic fibrous dysplasia) or multiple bones (polyostotic fibrous dysplasia). Fibrous dysplasia represents about 5% to 7% of benign bone lesions and monostotic fibrous dysplasia accounts for about 75% of the cases.^8^ Polyostotic fibrous dysplasia commonly occurs as part of the McCune-Albright syndrome with unilateral polyostotic fibrous dysplasia, ipsilateral café-au-lait spots, and endocrine disturbances such as precocious puberty.^8^ Patients with small, monostotic lesions may be asymptomatic, with osseous abnormalities usually identified incidentally on unrelated radiologic studies. Bone pain, swelling, and tenderness are common presentations in symptomatic patients.^9^ The most common sites of skeletal involvement include ribs, proximal femur, and craniofacial bones, typically the posterior maxilla as seen in this patient. Fracture is the most common complication and seen in more than half of patients with polyostotic form of the disease.^10^ Malignant transformation to osteosarcoma, fibrosarcoma, and chondrosarcoma occurs very infrequently, reportedly ranging from 0.4% to 4% and more common in craniofacial tumors.^10^ Histologically, fibrous dysplasia is composed of fibrous tissue with randomly oriented bony trabeculae that are thought to be formed by osseous metaplasia of the fibrous stroma.^10^

Incidentally discovered, asymptomatic, radiographically characteristic fibrous dysplasia lesions do not require further assessment, and follow-up radiographs every 6 months to look for new progression are recommended.^11^ In newly identified cases, a bone scan is needed to exclude a diagnosis of polyostotic disease. When polyostotic disease is found, referral to an endocrinologist for associated systemic abnormalities is warranted. Bisphosphonates have been utilized to decrease bone pain in symptomatic patients. Surgical procedures are required to correct deformities and prevent pathologic fractures. As in this patient, the growth of the tumor caused severe hyperglobus or proptosis of the left eye and a surgical procedure was performed to correct this malalignment.

## SUMMARY

A 57-year-old woman with a 15-year history of a slowly growing fibrous dysplastic maxillary bony tumor underwent total left maxillectomy with subsequent maxillary reconstruction with anterolateral thigh single perforator free flap and orbital floor reconstruction with a titanium mesh implant.
